# Clinical and sonographic impact of oral contraception in patients with deep endometriosis and adenomyosis at 2 years of follow-up

**DOI:** 10.1038/s41598-023-29227-2

**Published:** 2023-02-04

**Authors:** Pilar Carrillo Torres, M. Ángeles Martínez-Zamora, Cristina Ros, Mariona Rius, Eduard Mensión, Meritxell Gracia, Francisco Carmona

**Affiliations:** 1 Gynaecology Department Institute Clinic of Gynaecology, Obstetrics and Neonatology, Hospital Clinic of Barcelona, Universitat de Barcelona, Barcelona, Spain; 2 Gynaecology Department Institute Clinic of Gynaecology, Obstetrics and Neonatology, Hospital Clinic of Barcelona, Universitat de Barcelona, Institut d´Investigacions Biomèdiques August Pi i Sunyer (IDIBAPS), C/ Villarroel 170, 08036 Barcelona, Spain

**Keywords:** Endocrinology, Pathogenesis, Signs and symptoms, Reproductive biology, Reproductive disorders, Health care, Diagnosis, Medical imaging, Quality of life, Reproductive disorders

## Abstract

Nowadays, combined oral contraceptives (COCs) are successfully employed for the treatment of endometriosis (END) and adenomyosis (AD) in a large proportion of patients. However, literature focusing on the clinical and sonographic response to treatment in the long-term follow-up of patients with deep endometriosis (DE) and AD is scarce. The aim of this study was to evaluate the changes in the symptoms and the sonographic exams at 12 and 24 months of follow-up in patients who had received a flexible extended COC regimen containing 2 mg of dienogest/30 μg ethinyl estradiol. This prospective, longitudinal, observational study included women diagnosed with DE and AD presenting no surgical indication and were candidates to treatment with COCs. The presence and severity of dysmenorrhea, non-menstrual pelvic pain, deep dyspareunia, dyschezia and dysuria were evaluated using the Numerical Rating Scale (NRS) at baseline, and at 12 and 24 months of treatment. Transvaginal ultrasound was also performed at these check points searching for criteria of AD and reporting the size of the DE nodules and ovarian endometriomas (OE). Sixty-four patients were included. A significant decrease in the number of patients with severe dysmenorrhea and non-menstrual pelvic pain was reported during follow-up. The mean NRS score for dysmenorrhea, non-menstrual pelvic pain, deep dyspareunia, dyschezia and dysuria was also significantly lower at follow-up. There was a significant reduction in the sonographic number and type of AD criteria during follow-up after treatment. Similarly, a significant decrease in the size of OE and uterosacral ligament involvement in DE was observed at the 12-month follow-up, with a further, albeit not statistically significant, decrease in the 12- to 24-month follow-up. Additionally, torus and rectosigmoid DE decreased in size, although the reduction was not statistically significant at any study point. This prospective study suggests a clinical and sonographic improvement after a flexible extended COC regimen in DE and AD patients, which was significant at 12 months of follow-up. The improvement was more evident in AD and OEs compared with DE. Further research with a longer follow-up, larger sample size and comparison with other treatments is needed.

## Introduction

Endometriosis (END) and adenomyosis (AD) are benign, estrogen-dependent, chronic gynecological disorders which co-exist in approximately 35% of the patients’ sharing mechanisms of etiopathogenesis, clinical symptoms and treatment^1^.

The management of patients with END and/or AD includes both surgical and medical treatments^2^. The pharmacological approach aims to suppress ovulation and menstruation through hormonal treatments, with combined oral contraceptives (COCs) with low dose estrogen being considered as a first-line therapy, among other treatments^3–5^. COCs may be used in a conventional, continuous, or flexible extended regimen^3^. Extended regimens suppress ovarian function more reliably than 28-day cyclic regimens, with greater improvement of symptoms associated with menstruation^6,7^. In daily practice the use of a flexible extended regimen consisting in cycles of 120 consecutive days with active tablets followed by a 4-day tablet-free interval, either after 120 days or after 3 consecutive days of spotting^8^, has demonstrated to be beneficial due to a lower incidence of spotting and with a high rate of patient satisfaction^7^.

Most studies have analyzed the impact of COCs on pain symptoms in patients with different types of END post-surgical procedures^9–13^. However, there is scarce knowledge about the clinical and sonographic effects of COCs in patients with deep endometriosis (DE), which induces higher pain levels^14^, or in patients without previous surgery or in those with associated AD^15–18^. Furthermore, the few studies reporting the sonographic effects of these treatments are in isolated END patients, mostly with ovarian endometriomas (OE), and with follow-up periods shorter than 12 months^18–20^. To our knowledge, there are no previous reports on the sonographic changes after hormonal treatments in patients with DE and AD. This information is essential for adequate treatment and follow-up planning of these very common subgroups of patients.

Based on this scenario, the aim of this prospective observational study was to evaluate the changes occurring in the symptoms and in the sonographic exam reported at 12 and 24 months of follow-up by DE and AD patients who have received a flexible extended COC regimen.

## Materials and methods

### Study design

A prospective, single center, observational study was conducted at the Department of Gynecology of the Hospital Clinic of Barcelona, a tertiary university hospital in Spain and a referral center for the diagnosis and treatment of END and AD. The study was approved by the local Ethical Committee (*Comité Ético de Investigación con medicamentos del Hospital Clínic de Barcelona*), according to prevailing regulations (EMA/CHMP/ICH/135/1995). Written informed consent was obtained from all participants. All research was performed in accordance with relevant regulations and with the Declaration of Helsinki.

Patients who were not candidates for a surgical procedure were recruited in the outpatient clinic of our center and proposed to start treatment with COCs (2 mg dienogest/30 μg ethinyl estradiol) administered in a flexible extended regimen. None had contraindication or previous side effects for this type of treatment. A shared decision-making approach considering the individual preferences, side effects, individual efficacy, costs, and availability was carried out when counseling the patients in the choice between hormone treatments or surgical treatments for endometriosis-associated pain^21^.

The endpoints of this study were first, to study the changes in symptoms reported by patients. The Numerical Rating Scale (NRS) was used to evaluate pain (1 indicated absence of pain; 10 indicated the highest pain). Different types of pain were assessed: dysmenorrhea, non-menstrual pelvic pain, deep dyspareunia, dyschezia and dysuria and were determined at baseline and at 12 and 24 months of follow-up. Severe symptoms were considered with NRS scores ≥ 7^22^.

The second endpoint was to report the sonographic changes at the established check points. Changes were reported following the international group consensus guidelines performing high-resolution transvaginal ultrasound (TVUS)^23–26^.

The impact of the treatment was measured assessing the changes in the NRS for the pain parameters evaluated, as well as changes in the size of DE lesions and OE, when appropriate, and in the number and type of AD sonographic criteria present at baseline, and at 12 and 24 months of follow-up.

### Participants

Consecutive patients recently diagnosed with DE and AD, who were not candidates to surgical treatment (e.g. ureteral stenosis), were recruited between January 2019 and January 2020. Diagnosis was confirmed by a specialized sonographic exam.

To be eligible, the patients had to be > 18 years old, premenopausal women with both DE and AD and had to be candidates for treatment with a flexible extended COC regimen, consisting in cycles of 120 consecutive days with active tablets followed by a 4-day tablet-free interval, either after 120 days or after 3 consecutive days of spotting. They also had to agree to a clinical and sonographic evaluation at baseline, and at 12 and 24 months of follow-up.

**Figure 1:**
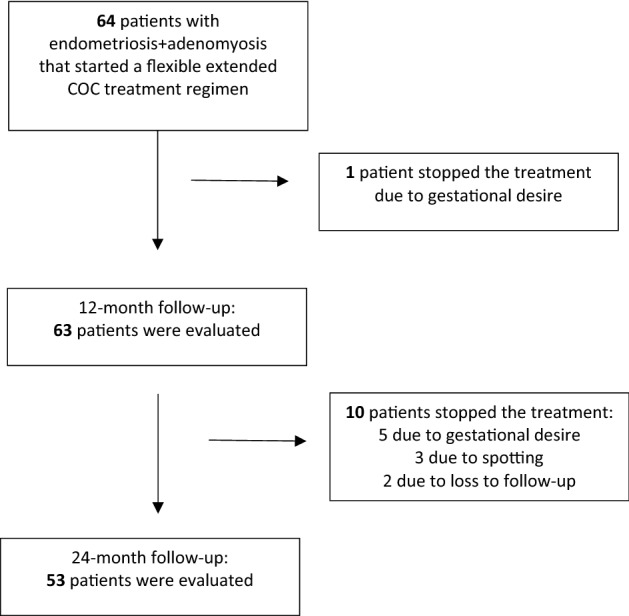
Flow chart of patient inclusion and drop-out.

The exclusion criteria for all participants included contraindications for estrogens and progestogens, psychiatric disorders, history of substance abuse, use of gonadotrophin-releasing hormone analogs or other hormonal treatments in the past 6 and 3 months, respectively, the presence of other uterine conditions (fibroids, polyps, and endometrial hyperplasia), neoplasms, and the presence of other chronic diseases such as heart disease and/or diabetes. Patients with surgical criteria or in whom TVUS was not possible (e.g. virgin patients) were excluded^25^. Sixty-four patients were eligible and recruited (Fig. 1).

### Study procedures

All patients underwent high resolution 2D-3D TVUS using an endovaginal probe (type RIC5-9, Voluson V730 Expert, GE Healthcare, Milwaukee, WI, USA) and received bowel preparation to improve DE identification and measurement, as previously described following the protocol of our center^25^. The exam was performed by two expert sonographers (CR, MR), who were not aware of the patient’s participation in the study, and the diagnoses of AD and END were established following the Morphological Uterus Sonographic Assessment (MUSA) group consensus^23^ for AD and the International Deep Endometriosis Analysis (IDEA) group consensus^24^ for END. Regarding AD, one point was given for the presence of each of the 6 following MUSA criteria: intramyometrial cysts, hyperechoic islands, fan-shaped shadowing, asymmetrical thickening, translesional vascularity and interrupted junctional zone^23^, requiring at least the presence of two criteria for establishing a diagnosis of AD^23,27^. The variables studied regarding END were the presence and size (mean of both maximum diameters, expressed in millimeters) of OE and rectosigmoid DE, uterosacral ligaments (USL) DE, torus, vaginal, ureteral, and vesical DE^24^.

### Statistical analysis

The sample size was estimated based on previous studies analyzing the impact of COCs on pain symptoms^15–18^ and sonographic changes^18–20^. The minimum number of patients needed to obtain an α error of 0.05 and a β error of 0.20 was estimated as 52. An additional 15% was allowed for possible dropouts. Categorical variables are expressed as count and percentages, and continuous variables as mean and standard deviation. The distribution of categorical variables was compared with the Chi-square test, and quantitative variables with the ANOVA test. Statistical significance was set at p < 0.05. The statistical analyses were performed with the Statistical Package for the Social Sciences software, release 25.0 for Windows (SPSS, Chicago, IL, USA).

### Ethics approval and consent to participate

The study was approved by the local Ethical Committee, according to prevailing regulations. Written informed consent was obtained from all participants.

## Results

### Patient characteristics

The baseline clinical characteristics and the types of END found in the sonographic evaluation are shown in Table 1. No patient had vesical, vaginal or ureteral DE. No adenomyomas were identified.

**Table 1:** Clinical characteristics of the enrolled population at baseline.

Variables	
Age	38.5 ± 4.8
BMI	22.3 ± 1.7
Smokers	10 (15.6%)
Nulliparity	45 (70.3%)
Infertility	32 (50%)
Adenomyosis	63 (100%)
Ovarian endometriomas	40 (63.5%)
Rectosigmoid DE	30 (47.6%)
Torus DE	36 (57.1%)
USL DE	24 (38.1%)

Results are expressed as number and percentage or mean ± standard deviation.

*DE* deep endometriosis, *BMI* body mass index, *USL* uterosacral ligaments.

At the 12-month follow-up, 63/64 patients remained on the treatment, and the only drop-out was due to gestational desire. Fifty-three patients completed the 24-month treatment. The reason for early treatment discontinuation by 10 patients was gestational desire (n = 5), persistent spotting (n = 3) and loss to follow-up (n = 2) (Fig. 1). No serious adverse events were reported during the study period. Non-serious adverse effects included headache and breast tension (not requiring treatment discontinuation). None of the patients required surgery during the study period.

### Effect of COCs in the clinical evaluation

As expected, there was a decrease in the number of patients with severe dysmenorrhea (n = 52 (82.5%); vs. n = 4 (6.3%); vs. n = 0 (0%) [p < 0.001]) and non-menstrual pelvic pain (n = 20 (31.7%); vs. n = 0 (0%), vs. n = 0 (0%) [p < 0.001]) from baseline to the 12- and 24-month follow-ups. The mean NRS for dysmenorrhea, non-menstrual pelvic pain, deep dyspareunia, dyschezia and dysuria also decreased in the 12- and 24-month follow-ups (Table 2).

**Table 2:** Effect of COCs on symptomatic evaluation at baseline and at 12 and 24 months of follow-up in endometriosis and adenomyosis patients.

Variables	Baseline^a^ N = 64	12-month follow-up N = 63	24-month follow-up N = 53	p-value
Severe dysmenorrea*	52 (82.5%)^a^	4(6.3%)^b^	0 (0%)^c^	^a-b^p < 0.001;^b-c^ NS
				^a-c^p < 0.001
Severe non-menstrual pelvic pain*	20 (31.7%)^a^	0 (0%)^b^	0 (0%)^c^	^a-b^ p < 0.001;^b-c^ NS
				^a-c^p < 0.001
Dysmenorrhea**	8.05 ± 1.61^a^	3.76 ± 2.19^b^	1.51 ± 1.60^c^	^a-b, b-c, a-c^p < 0.001
Non-menstrual pelvic pain**	5.95 ± 1.52^a^	2.08 ± 1.65^b^	0.79 ± 1.17^c^	^a-b, b-c, a-c^ p < 0.001
Dyspareunia**	3.70 ± 3.59^a^	0.81 ± 1.85^b^	0.70 ± 1.65^c^	^a-b, a-c^p < 0.001
				^b-c^NS
Dyschezia**	1.98 ± 3.41^a^	0.44 ± 1.39^b^	0.45 ± 1.42^c^	^a-b, a-c^p < 0.001
				^b-c^NS
Dysuria**	0.43 ± 1.68^a^	0.11 ± 0.63^b^	0.13 ± 0.68^c^	^a-b, b-c, a-c^p = NS

Results expressed as number and percentage or mean ± standard deviation.

*NS* not significant.

*Numerical Rating Scale (NRS) ≥ 7.

**Mean NRS.

### Effect of COCs on the sonographic expression of adenomyosis

A significant reduction in the sonographic expression of AD was observed when comparing baseline results to the 12- and 24-month follow-ups, with a decrease in the number of patients fulfilling this diagnosis (n = 63 (100%) vs. n = 42 (66.7%) vs. n = 32 (60.4%), respectively (p < 0.001)). In addition, the number of patients who met 4 and 3 criteria decreased at 12 and 24 months (p < 0.0001) (Table 3).

**Table 3:** Number of adenomyosis diagnostic sonographic criteria at baseline and at 12 and 24 months of follow-up.

	Baseline^a^ N = 64	12-month follow-up^b^ N = 63	24-month follow-up^c^ N = 53	p-value
> 2 criteria	63 (100%)^a^	42 (66.7%)^b^	32 (60.4%)^c^	^a-b, a-c^ p < 0.001
				^b-c^ NS
> 3 criteria	58 (92.1%)^a^	33 (52.4%)^b^	23 (36.5%)^c^	^a-b, a-c^ p < 0.001
				^b-c^ p = NS
> 4 criteria	48 (76.2%)^a^	18 (27.5%)^b^	13 (20.7%)^c^	^a-b, a-c^ p < 0.001
				^b-c^ p = NS

Results expressed as number and percentage or mean ± standard deviation.

*NS* not significant.

**Figure 2:**
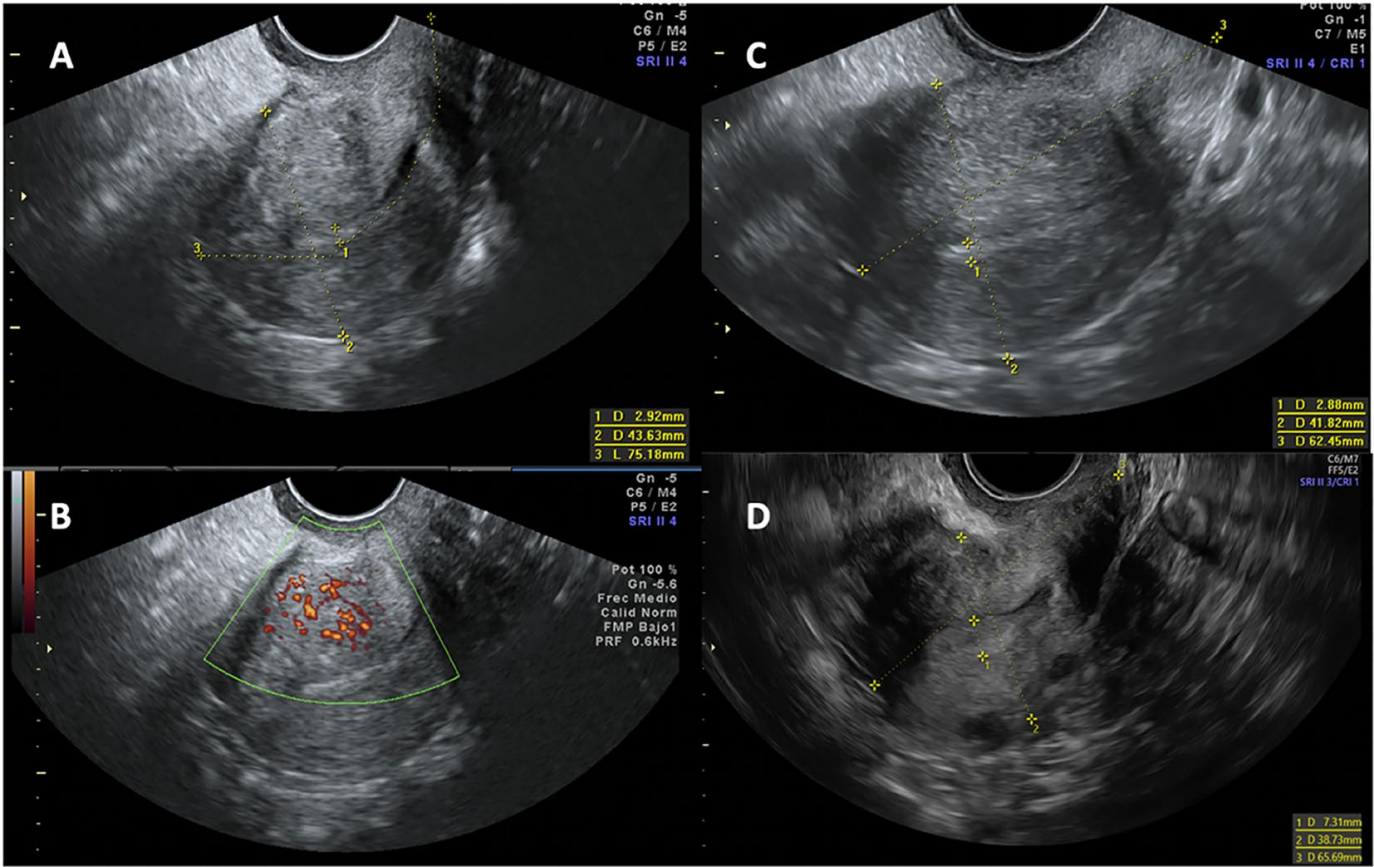
Adenomyosis sonographic evolution at baseline and at 12 and 24 months of follow-up. (**A** and **B**) Baseline ultrasound with the presence of 5 adenomyosis criteria; (**A**) Hyperechoic islands, fan-shaped shadowing, uterine wall asymmetrical thickening, interrupted junctional zone and (**B**) Translesional vascularity; (**C**) 12-month follow-up with hyperechoic islands and uterine wall asymmetrical thickening as mild signs of adenomyosis. (**D**) 24-month follow-up with no signs of adenomyosis.

Hyperechoic islands and an interrupted junctional zone were the most frequent AD criteria found at baseline and at the subsequent study points. All criteria decreased during follow-up, with the reduction between baseline and the 12-month follow-up being statistically significant (Table 4, Fig. 2).

**Table 4:** Adenomyosis diagnostic sonographic criteria at baseline and at the 12 and 24-month follow-ups.

	Baseline^a^ N = 64	12-month follow-up^b^ N = 63	24-month follow-up^c^ N = 53	p-value
Hard criteria				
Intramyometrial cysts	22 (34.4%)^a^	10 (15.9%)^b^	7 (13.2%)^c^	^a-b, a-c^p < 0.0001
				^b-c^p = NS
Hyperechoic islands	62 (96.9%)^a^	38 (60.3%)^b^	29 (54.7%)^c^	^a-b, a-c^p < 0.0001
				^b-c^NS
Soft criteria				
Fan-shaped shadowing	44 (68.7%)^a^	23 (36.5%)^b^	17 (32.1%)^c^	^a-b^p = 0.0004
				^a-c^p < 0.0001
				^b-c^p = NS
Asymmetrical thickening	45 (70.3%)^a^	22 (34.9%)^b^	18 (33.4%)^c^	^a-b, a-c^p < 0.0001
				^b-c^p = NS
Translesional vascularity	30 (46.9%)^a^	11 (17.5%)^b^	6 (11.3%)^c^	^a-b^p = 0.0006
				^a-c^p < 0.0001
				^b-c^NS
Interrupted junctional zone	57 (89.1%)^a^	36 (57.1%)^b^	25 (47.2%)^c^	^a-b, a-c^p < 0.0001
				^b-c^p = NS

Results expressed as number and percentage.

*NS* not significant.

### Effect of COCs on the sonographic expression of endometriosis

**Figure 3:**
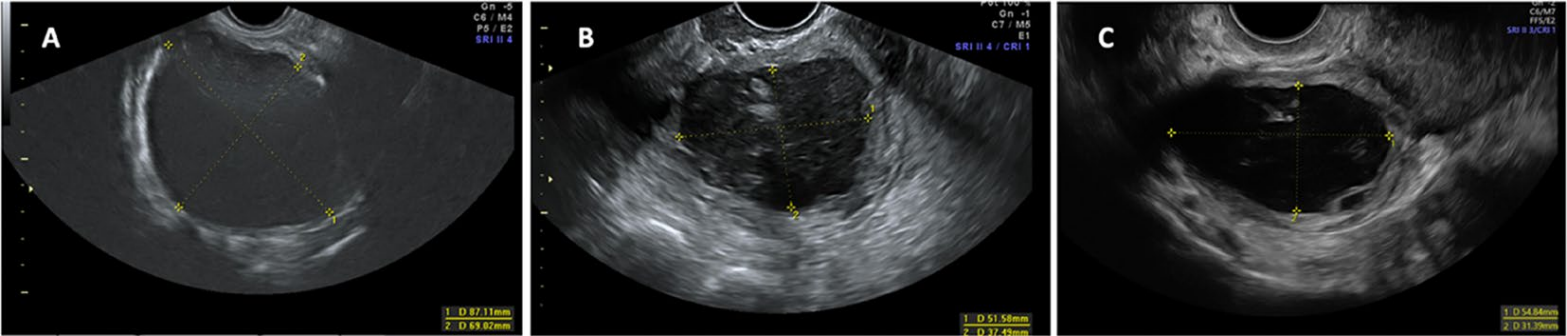
Sonographic evolution of ovarian endometrioma at baseline and at 12 and 24 months of follow-up. (**A**) Large endometrioma with a mean size of 78.0 mm which decreased to 44.5 mm at the 12-month follow-up in image (**B**) and to 43.1 mm at the 24-month follow-up in image (**C**).

**Figure 4:**
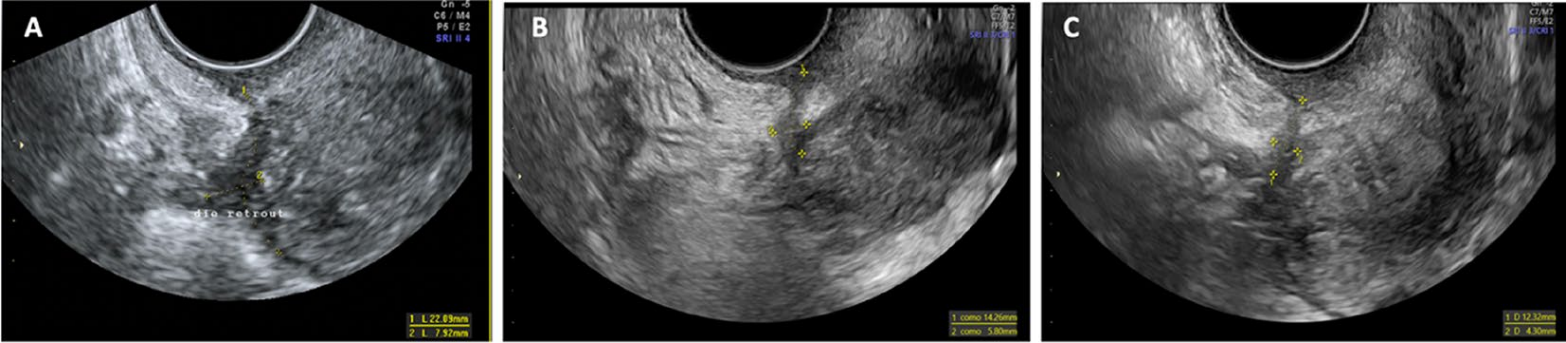
Sonographic evolution of uterosacral deep endometriosis at baseline and at the 12- and 24-month follow-ups. Image (**A**) Uterosacral deep infiltrating nodule with a mean size of 15.0 mm which decreased to 10.0 mm at the 12-month follow-up in image (**B**) and to 8.3 mm at the 24-month follow-up in image (**C**).

We found a significant reduction in OE size when comparing baseline to the 12- and 24-month follow-ups (33.45 ± 16.83 mm vs. 22.67 ± 4.02 [p < 0.02] vs. 18.62 ± 13.48 mm [p < 0.01], respectively) (Table 5, Fig. 3). A decrease in the number of patients with torus DE, USL DE and rectosigmoid DE at 12 and 24 months was observed, although it was not statistically significant. The size of USL significantly decreased from baseline to 12 and 24 months of follow-up (20.91 ± 8.75 mm; vs. 15.48 ± 6.07 [p < 0.03]; vs. 13.13 ± 4.48 mm [p < 0.004], respectively) (Table 5, Fig. 4).

**Table 5:** Effect of COCs on the sonographic expression of endometriosis at baseline and at 12 and 24 months of follow-up.

Measurement	Baseline^a^ N = 64	12-month follow-up^b^ N = 63	24-month follow-up^c^ N = 53	p-value
Ovarian endometrioma	33.45 ± 16.83^a^	22.67 ± 14.02^b^	18.62 ± 13.48^c^	^a-b^p < 0.02
				^b-c^p = NS
				^a-c^ p < 0.01
Torus DE	25.78 ± 12.98^a^	20.64 ± 8.35^b^	19.53 ± 8.62^c^	^a-b; b-c, a-c^p = NS
USL DE	20.91 ± 8.75^a^	15.48 ± 6.07^b^	13.13 ± 4.48^c^	^a-b^p < 0.03;^b-c^ p = NS
				^a-c^p < 0.004
Rectosigmoid DE	24.33 ± 10.23^a^	22.22 ± 8.39^b^	20.96 ± 8.77^c^	^a-b; b-c, a-c^p = NS

Results expressed as mean ± standard deviation.

Measurement is expressed in millimeters.

*USL* uterosacral ligaments, *DE* deep endometriosis, *NS* not significant.

## Discussion

Management of END and AD is challenging for gynecologists, considering that the therapeutic strategy should be modulated and tailored to patient characteristics. Our study provides valuable information about the clinical and sonographic changes of DE lesions and AD criteria in patients with both entities. It is noteworthy that in a high percentage of patients DE is associated with AD, and therefore, it is important to know if hormonal treatments are effective in patients with both entities in the long-term to better schedule visits and radiological follow-up. Pharmacological treatments, such as COCs, could be a feasible option in a large number of these patients^15–18^. COCs have been used in the treatment of pelvic pain in patients with END for more than 50 years and have been reported to reduce or eliminate pain in approximately 90% of the patients^2^. In the present study, a flexible extended COC regimen was implemented instead of a conventional regimen, since previous research and our large institutional experience have shown that this regimen provides better control of pain symptoms and spotting bleeding patterns are better controlled by patients^5^. However, the most effective combination of estroprogestagens is unknown. Regarding the progestogens contained in the COCs, studies have proven the health benefits of fourth generation progestogens such as drosperinone and dienogest^28^. We used the combination or 2 mg dienogest and 30 μg ethinyl estradiol due to its low cost, in addition to being a well-tolerated progestogen with few side effects in most patients.

The main objective of this study was to evaluate the effects of an extended long-term COC regimen on different pain symptoms frequently reported by patients with END and/or AD, and we observed a reduction in the number of patients with severe dysmenorrhea and non-menstrual pelvic pain during follow-up. The mean NRS for dysmenorrhea, non-menstrual pelvic pain, deep dyspareunia, dyschezia and dysuria were also lower in the 12- and 24-month follow-ups. These findings are in concordance with previously published studies^3,5,9–13,15,16^ although our research provides one of the longest follow-ups.

It was also our goal to investigate the effects of COCs on the features of DE and AD in the TVUS exam in a minimum two-year follow up. A reduction in the sonographic expression of both END and AD was observed. It is important to stress that a significant decrease in the size of USL was noted on comparing baseline values to the 12-month follow-up, with this decrease showing a trend to being even higher at 24 months. In addition, torus and rectosigmoid DE lesions showed a reduction, albeit not statistically significant, in size. The decrease in OE size was significant and more remarkable compared to DE lesions and was also significant only at the first study point one year after starting treatment. The predominance of fibrosis and the presence of fewer endometrial glands in DE lesions may be responsible for a lower sonographic response in some types of DE lesions compared to other types of END such as OE^29–31^. These findings in the TVUS exam were also consistent with previous reports. Other authors have described a statistically significant reduction in the volume of OE after different types of hormonal treatment such as dienogest^17–20^ or COCs^3,5,32^. Few studies evaluating the sonographic changes in isolated DE rectosigmoid endometriotic nodules after multiple types of hormonal treatments^33–36^ observed a significant reduction of rectosigmoid endometriosis nodules at 6 and 12 months of follow-up. To our knowledge, this issue has not previously been analyzed with a longer follow-up. Furthermore, no previous study has evaluated the sonographic impact of COCs on AD. It is noteworthy that there was a significant reduction in the number of AD criteria in the 12- and 24-month follow-ups and that almost 40% of patients who showed AD at baseline had no AD findings in the 24-month follow-up thereby showing complete sonographic response following hormonal treatment. Similarly to USL DE and OE, there was a significant decrease of all sonographic AD criteria at 12 months of follow-up and a trend to a decrease until 24 months of follow-up. Furthermore, previous studies have suggested that endometriosis is a disease that probably progresses from adolescence until adulthood^37,38^, whereas the highest prevalence of deep infiltrating lesions is observed after the age of 26 years and appears to increase from adolescence until the fourth decade. Therefore, these authors suggested that policies relating to the prevention and early diagnosis of endometriosis should focus on women younger than 25 years and stressed that the progression of endometriosis lesions in older patients still in the fertile period may have ceased^37,38^. This may have occurred in a percentage of patients with a mean age of 38.5 ± 4.8 years in our study, but this issue could not be ascertained in our study as a control group without medical treatment was not included and further research is warranted to clarify this^37,38^.

The strengths of this study include the strict inclusion and exclusion criteria, with our long-term follow-up being one of the longest reported in the literature. Other strengths were the assessment of the clinical presentations of END in all its forms (dysmenorrhea, non-menstrual pelvic pain, deep dyspareunia, dyschezia and dysuria), evaluation of both AD and DE and their sonographic expression and the use of the same COCs in all patients.

Several limitations of our study should be considered for data interpretation: first, we enrolled a relatively small number of patients, although comparable or even superior to other previously published studies and according to sample size estimation^17,19,32–36^. Second, we evaluated patients at two arbitrary study points, at 12 and 24 months of follow-up, but as the most important changes were observed at one year of follow-up, it would have been interesting to evaluate the changes before that study point and later than 24 months to reevaluate the improvement with a shorter and longer follow-up. Third, our study population did not have all the locations of DE described, but did have the most common, and therefore, vesical, vaginal or ureteral endometriosis DE should be evaluated in another affected population. Finally, we did not compare the results of different COC regimens, other hormonal treatments or the use of analgesics. Regarding this last issue, although we do not have precise information on the dosage of concomitant analgesics used by patients at diagnosis and follow-up, the reduction in pain symptoms at 12 and 24 months suggests a reduced intake of painkillers.

Based on this scenario, future research should consider the design of randomized controlled trials in a large population with a longer follow-up with more study points. Moreover, the comparison of different doses and types of hormones should be analyzed.

## Conclusions

To conclude, this prospective study suggests a significant reduction in pain symptoms and sonographic expression of END and AD findings after a flexible extended COC regimen at 12 months of follow-up, with a trend to improvement at between 12 and 24 months of follow-up. The improvement was more evident in AD, USL DE and OE compared with torus and rectosigmoid DE. Further research with a longer follow-up, larger sample size and comparison with other treatments is needed.

## Data Availability

The datasets used and/or analyzed during the current study are available from the corresponding author on reasonable request.
